# Social justice, access and quality of healthcare in an age of austerity: users’ perspective from rural Iceland

**DOI:** 10.1080/22423982.2017.1347476

**Published:** 2017-08-01

**Authors:** Sonja S. Gustafsdottir, Kristjana Fenger, Sigridur Halldorsdottir, Thoroddur Bjarnason

**Affiliations:** ^a^ Faculty of Occupational Therapy, School of Health Sciences, University of Akureyri, Akureyri, Iceland; ^b^ Head of Faculty of Graduate Studies, School of Health Sciences, University of Akureyri, Akureyri, Iceland; ^c^ Faculty of Social Sciences, School of Humanities and Social Sciences, University of Akureyri, Akureyri, Iceland

**Keywords:** Rural communities, health care services, social justice, equity in health, access to health care, human rights, mixed methods, transformative design, users’ perspective, Iceland

## Abstract

Iceland is sparsely populated but social justice and equity has been emphasised within healthcare. The aim of the study is to examine healthcare services in Fjallabyggð, in rural northern Iceland, from users’ perspective and evaluate social justice, access and quality of healthcare in an age of austerity. Mixed-method approach with transformative design was used. First, data were collected with questionnaires (response rate of 53% [N=732] in 2009 and 30% [N=415] in 2012), and analysed statistically, followed by 10 interviews with healthcare users (2009 and 2014). The results were integrated and interpreted within Bronfenbrenner’s Ecological Model. There was significantly less satisfaction with accessibility and variety of healthcare services in 2012 after services downsizing. Solid primary healthcare, good local elderly care, some freedom in healthcare choice and reliable emergency services were considered fundamental for life in a rural area. Equal access to healthcare is part of a fundamental human right. In times of economic downturn, people in rural areas, who are already vulnerable, may become even more vulnerable and disadvantaged, seriously threatening social justice and equity. With severe cutbacks in vitally important healthcare services people may eventually choose to self-migrate.

## Introduction

Equity in health and equal access to health care are widely acknowledged goals of health policy in most industrialised countries [1]. It is one of the key principles of the Ottawa Charter and should be achieved by means of actions aimed at reducing differences and ensuring equal opportunities for people to achieve their fullest health potential [2,3]. As an ethical concept equity is closely related to the fundamental human right to the highest attainable standard of health [2,4]. Equity is not something that is achieved once and for all; it is a moral imperative and an important indicator of the quality of countries and communities [5,6].

There are considerable health inequalities within and between countries in Europe [7]. Such inequities within countries are the major focus of the WHO call for social justice, “Health for All” [8] and the new WHO European policy for health, “Health 2020” [9]. There is clear evidence that modern health care services influence life expectancy and morbidity, and traditionally disadvantaged groups have reduced access to interventions known to be effective [10]. Access is multifaceted, measuring not only characteristics of the health care system, but in addition characteristics of the individual and the areas the relevant health care system serves [11].

Many European governments have responded to the economic difficulties of recent years by imposing strict fiscal austerity, in particular in the sectors of health and welfare [12]. In Iceland, public expenditure on health care was reduced by 24% in the aftermath of the collapse of the entire national banking sector in 2008. Iceland has a Nordic social welfare system that provides health care, which is paid mostly by taxes and to a lesser extent by service fees [13]. In 2011, a 9% share of the country’s GDP expenditure was put on health compared with an average of 9.3% in OECD countries [14].

As a part of these austerity measures, the Icelandic health care system was restructured in order to create fewer and stronger health care regions across the country. Iceland is sparsely populated, with about 330,000 inhabitants in an area of 103,000 km^2^. The capital area is home to about two-thirds of the population. The remaining third lives around the coastline, while the interior is uninhabited and is mainly characterised by sand and lava fields, rivers, mountains and glaciers. The aim was to provide equal or better quality health care in rural areas at lower costs through institutional mergers and expanded health care regions. While costs have been successfully lowered, it is unclear to what extent rural residents feel that their access to high-quality and diverse health care is equal or better than before.

### The case of the rural Fjallabyggð area in northern Iceland

According to Icelandic law, equal access to health care is a human right that everyone should have irrespective of place of residence. This means that Iceland’s health care legislation emphasises the right of all citizens to the best health care available [15], thus declaring the intentions of prioritising social equality in health. However, it is known that residents in rural areas in Iceland constitute a vulnerable population that report poorer self-rated health and yet have lower prevalence of diagnosed diseases, perhaps due to selective migration of those with chronic diseases [16]. Migration may, however, lead to cultural bereavement, changes in identity and concept of self as well as the loss of important social support systems [17].

The municipality of Fjallabyggð in northern Iceland provides a unique case for studying consolidation in the health care system in an age of austerity. It has a population of little over 2,000 inhabitants and was established in 2006 with the merger of 2 relatively isolated fishing towns in northern Iceland, Ólafsfjörður and Siglufjörður, in anticipation of the opening of a set of 2 road tunnels in 2010, connecting the 2 towns with a 17 km road.

**Figure 1.**
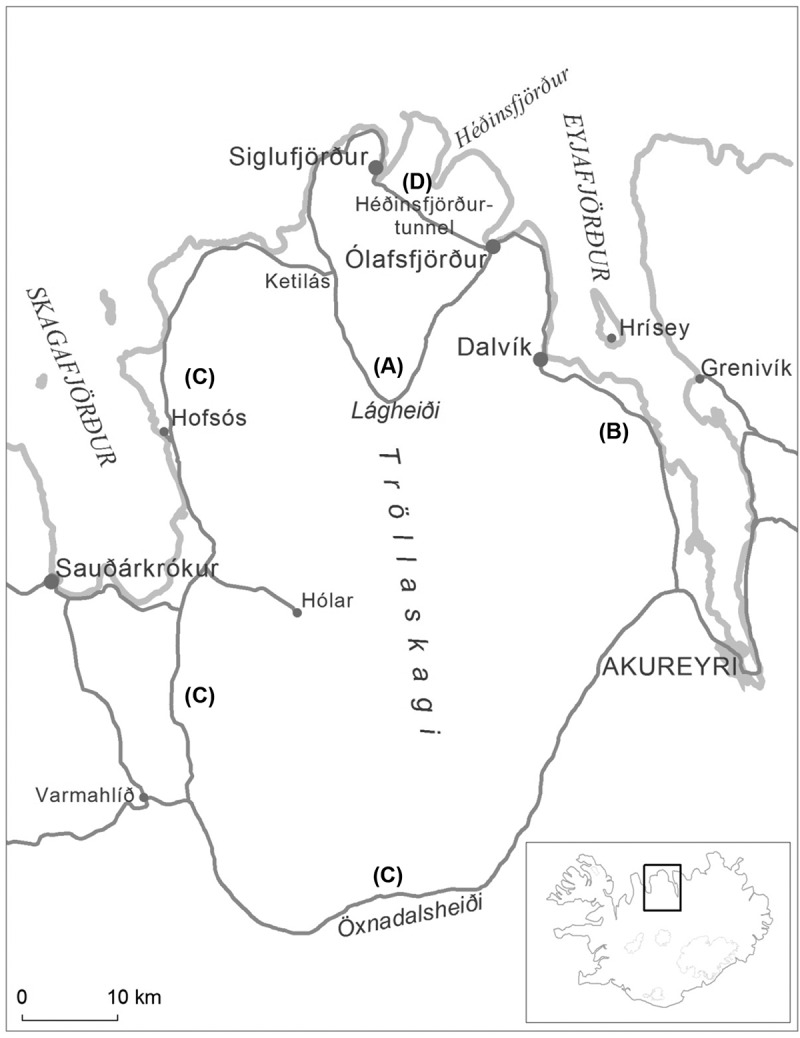
Map of Fjallabyggð and the surrounding area in northern Iceland. (A) 62 km gravel road connecting Ólafsfjörður and Siglufjörður. (B) 61 km national highway connecting Ólafsfjörður and the regional centre of Akureyri. (C) 192 km national highway connecting Siglufjörður and the regional centre of Akureyri. (D) 17 km road and tunnels connecting Ólafsfjörður and Siglufjörður.

Figure 1 shows the location of the 2 fishing towns and the study area in northern Iceland. Prior to the opening of the tunnels, the summer road between the 2 towns was 62 km via a gravel road over the mountain pass of Lágheiði (A). The regional centre of Akureyri was 61 km from Ólafsfjörður via the national highway (B) and thus 123 km from Siglufjörður when the mountain pass was open during the summer. When the mountain pass closed during the winter, however, Siglufjörður was 192 km from the regional centre via the national highway (C) and 233 km from neighbouring Ólafsfjörður by the 2 highways (B and C).

The Héðinsfjörður tunnels between the 2 towns transformed the transportation landscape of the area. The 2 towns were connected all year with a 17 km highway (D) via a 4 km tunnel, a 7 km tunnel and a total of 6 km connecting roads. While the distance between Ólafsfjörður and Akureyri was not affected after the opening of the tunnels, the town of Siglufjörður was 77 km from Akureyri in winter as well as summer. There is limited public transportation in the area but walking distances are short within each of the 2 towns.

During the planning phase of the tunnel project, it was made clear that the transformation of the transportation landscape would lead to a restructuring of municipal and state services [18]. Residents were nevertheless assured that such restructuring would lead to improved services with unchanged funding, rather than unchanged services with less funding. During the construction phase, however, the Icelandic banking system collapsed in October 2008 and drastic national austerity measures were enforced at both the state and municipal level. These infrastructure improvements thus at best counteracted severe cuts in local services by providing substantial new opportunities for quality health care. The 23% cutbacks in public health care expenditure in Fjallabyggð between 2007 and 2011 were close to the national average, but the 20% cut in public expenditure for care of the elderly in Fjallabyggð was substantially above the national average of 10% [19]. According to the local authorities, health care staff was also reduced between the years 2009 and 2012 by 5.9 full-time equivalent positions, from 51.1 to 45.2, which resulted in fewer services [19].

Research from the users’ perspective can strengthen health care services by giving them a voice and thereby empowering them to criticise and affect the available services [20,21]. A sustained conversation with users of services has been argued to be among the civil rights of users [22]. Users’ perspectives on health care in rural areas are largely missing in Iceland. The aim of the present study was to examine health care services in Fjallabyggð from the users’ perspective, regarding access, variety and quality of health care services, with the emphasis of evaluating social justice and equity in the provision of health care in this rural area.

## Materials and methods

**Figure 2.**
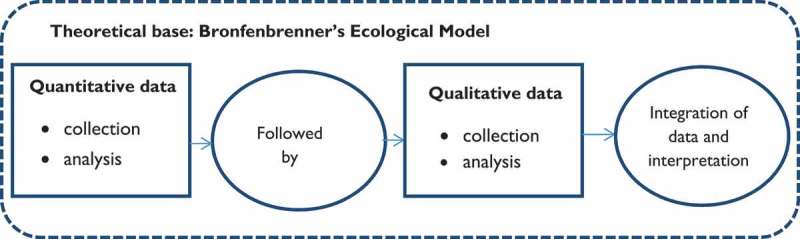
Procedural diagram for the “mixed-method transformative design”.

We used a “mixed-methods transformative design” (Figure 2) which is often used when social inequality and status of minority groups is being studied with respect to culture and social influences as well as the perspectives of inhabitants [23]. In the present context, we are looking at major social changes and downsizing of health care in a rural area and the perspectives of inhabitants regarding, for example, alternation of access to health care.

### Data collection and analysis

Quantitative data were collected and analysed, followed by qualitative data collection and analysis. Subsequently, the results were integrated and interpreted within the framework of Bronfenbrenner’s Ecological Model. The model is based on the understanding that there are interactive effects between the individual and the environment regarding 5 distinct levels: the microsystem, the mesosystem, the exosystem, the macrosystem and the chronosystem [24]. Therefore, the different levels of the environment in Fjallabyggð, where the inhabitants have been subjected to many diverse social changes, both positive and negative, were examined.

Table 1 shows the distribution of the population of the municipality of Fjallabyggð at the time of the 2 surveys by town, gender, and age groups. The town of Siglufjörður is slightly larger than Ólafsfjörður, but the population of both towns declined by about 6% between the 2 surveys. As in many such rural towns, there are more men than women. However, the gender imbalance was reduced from 888 to 959 women per 1,000 men between 2009 and 2012 as the number of men declined at a slightly higher rate. A relatively large proportion of the population is over the age of 40, but the largest decline between 2009 and 2012 was 9% in the age group 41–66 years old.

**Table 1.** Age and gender distribution of inhabitants in 2009 and 2012.

	2009	2012	Change (%)
*Town within Fjallabyggð*			
Siglufjörður	1,277	1,203	6
Ólafsfjörður	850	799	6
*Gender*			
Male	1,126	1,022	9
Female	1,001	980	2
*Age groups*			
0–17 years old	445	428	4
18–40 years old	552	524	5
41–66 years old	749	684	9
67 years or older	381	366	4
***Total***	***2,127***	***2,002***	***6***

Resident surveys were conducted in-home in October and November 2009 before the opening of the tunnels and in October and November 2012 after the opening. All residents 18 year and older were asked to respond to a standardised 10-page questionnaire examining, for example, people’s perspectives regarding access, variety and quality of health care services. In 2009, the questionnaires were distributed and collected by research assistants, but in 2012, respondents could either send their questionnaire in a pre-stamped envelope or answer the survey electronically.

A total of 732 questionnaires were completed in 2009 and 416 in 2012. Based on the census conducted as part of the project, the estimated response rate was 53% in 2009 and 30% in 2012. Respondents in the age group 18–25 were 9–11% of the samples in 2009 and 2012, compared with 17–19% in the target population. This is in line with research demonstrating that Statistics Iceland overestimates the actual number of young people in that age group in Fjallabyggð. This is primarily because frequently young people studying elsewhere are still registered in their community of origin. The respondents to the surveys did not differ significantly in terms of gender, residence, education, employment status or living arrangements. However, the proportion aged 67 years or older was significantly higher in 2012.

Table 2 shows an overview of the survey data used in the current study. Perceptions of health care were measured by: “How satisfied or dissatisfied are you with the following aspects of health services in Fjallabyggð?”. The 3 sub-questions were “Access to health services”, “Variety of health services” and “Quality of health services”. The categories were “Very dissatisfied”, “Rather dissatisfied”, “Neither nor”, “Rather satisfied” and “Very satisfied”.

**Table 2.** Descriptive statistics for statistical analysis.

	Range	2009 (s.e.)	2012 (s.e.)
*Town within Fjallabyggð*			
Siglufjörður	0–1	.60 (.02)	.63 (.02)
Ólafsfjörður	0–1	.40 (.02)	.37 (.02)
*Gender*			
Male	0–1	.48 (.02)	.48 (.02)
Female	0–1	.52 (.02)	.52 (.02)
*Age at survey*			
18–25 years old	0–1	.09 (.01)	.06 (.01)
26–40 years old	0–1	.17 (.01)	.13 (.02)
41–66 years old	0–1	.54 (.02)	.54 (.02)
67 years or older	0–1	.20 (.01)	.27 (.02)
*Living arrangements*			
Does not live alone	0–1	.87 (.01)	.86 (.02)
Lives alone	0–1	.13 (.01)	.14 (.02)
*Education*			
Trade certificate	0–1	.25 (.02)	.28 (.02)
University degree	0–1	.11 (.01)	.14 (.02)
Other education	0–1	.64 (.02)	.58 (.02)
*Employment status*			
Not employed	0–1	.30 (.02)	.35 (.02)
Employed	0–1	.70 (.02)	.65 (.02)
*Perceptions of health care*			
Access to health care	1–5	4.29 (.03)	4.12 (.04)
Variety of health care	1–5	3.80 (.03)	2.68 (.05)
Quality of health care	1–5	3.97 (.03)	3.97 (.04)
N (listwise)	732	416	

To ensure anonymity in a population survey of these 2 small towns, respondents were asked about their age in 4 broad age categories; 18–25 years old, 26–40 years old, 41–66 years old and 67 years or older. They were also asked about other people living in the same household, including parents, siblings, spouses, children and other relatives or non-relatives. The responses were recoded to identify those who lived alone. Furthermore, respondents were asked about their education, including primary and secondary education, trade certification and university education. The responses were recoded to identify those with trade certification or a university degree. Respondents were finally asked about their labour market participation in 3 categories: employed full-time, employed part-time and not employed. The responses were recoded to identify those who were not employed.

Permission for the qualitative part was obtained from the National Bioethics Committee (VSNb2010060014/03.1). The aim of the interviews was to deepen the information gathered in the quantitative part of the project and to gain an understanding of users’ perspective on healthcare services. Through purposeful sampling, participants from both towns, of both sexes, and of different stages in life were interviewed by the first 2 authors in 2009 and 2014. Age of respondents was 24–74 years. Length of the 10 interviews ranged from 30 to 60 minutes. The interviews were tape-recorded and transcribed verbatim. The 3 first authors each coded and categorised the data, compared and came to a consensus.

## Results

**Figure 3.**
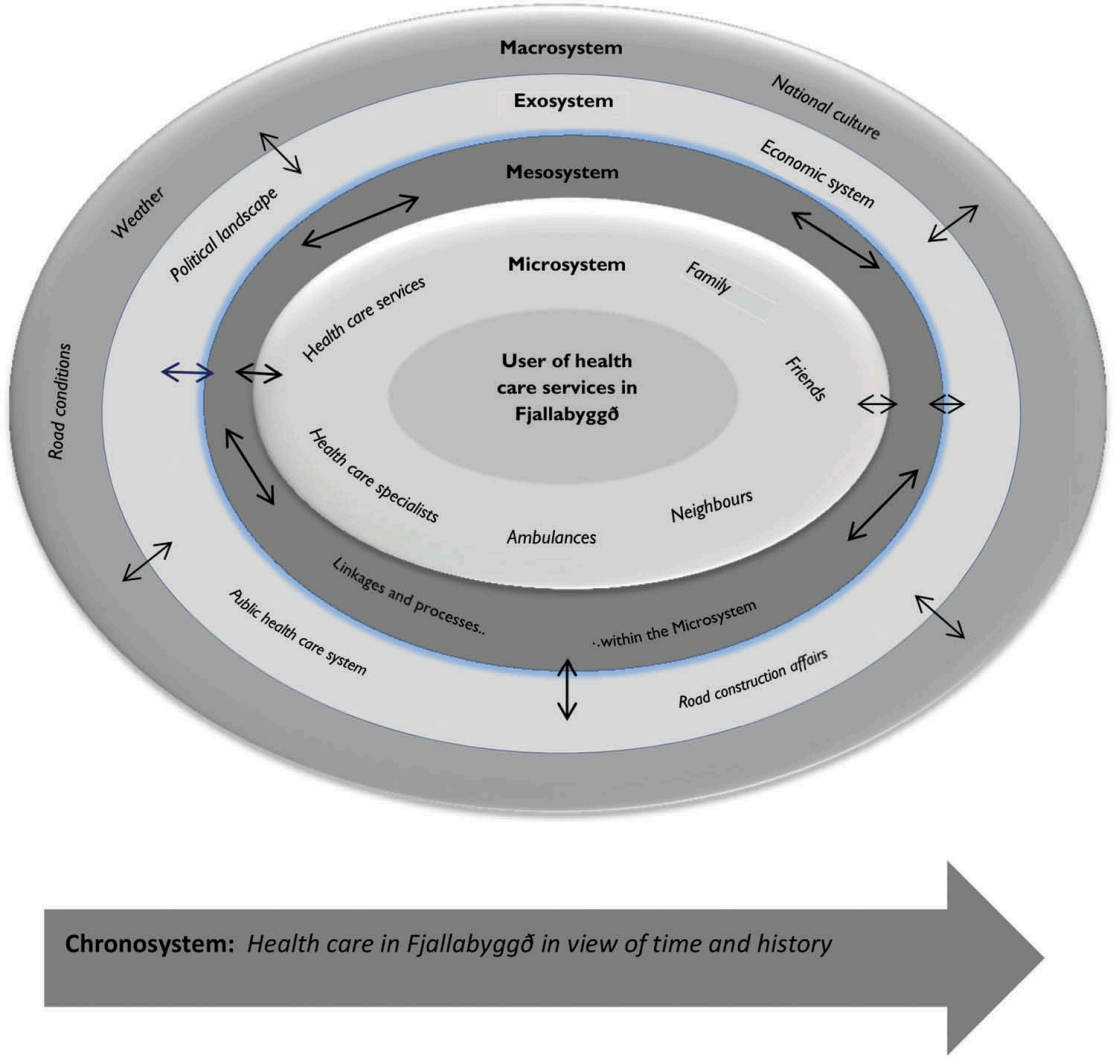
Reciprocal impact of environmental factors and the perspective of health care users.

A variety of environmental factors influences the perspective of the residents of Fjallabyggð regarding health care services and their interpretation of own experience. Figure 3 portrays the reciprocal impact of environmental factors and the perspective of health care users regarding health care services based on Bronfenbrenner’s model [24]. Changes in one system can affect another. As an example, the changes in road connections, the merger of health care institutions and cutbacks in health care services due to societal economy will affect individuals and their immediate environment. The aim of the model is to provide an overview of the changes that occurred in the environment and their influences on the individual.

### The results of the surveys

Satisfaction with *accessibility* and *variety* of health care services appears to have declined significantly in Fjallabyggð between 2009 and 2012. A total of 65% of the respondents were rather or very satisfied with the *variety* of health care services in 2009, compared with 61% in 2012. Significantly fewer respondents were very satisfied with the variety of health care services in 2012 (χ^2^=10.0; df=4, p<0.05). The results of the multivariate analyses shown in Table 2 demonstrate that the decrease in satisfaction with the diversity of health care services remains statistically significant after controlling for differences in satisfaction between men and women, age, living arrangements, education, employment status and town of residence.

A total of 89% were rather or very satisfied with the *accessibility* of health care services in 2009, compared with 85% in 2012. However, significantly fewer respondents were *very* satisfied with their access to health care in 2012 (χ^2^=27.4; df=4, p<0.001). The multivariate results in Table 3 show that the decrease in satisfaction with access to health care remains statistically significant after controlling for differences in satisfaction between men and women, age, living arrangements, education, employment status and town of residence.

**Table 3.** Predictors of perceived access, variety and quality of health care in Fjallabyggð 2009 and 2012.

	Access to health care	Variety of health care	Quality of health care
*Town within Fjallabyggð*			
Siglufjörður	Contrast	Contrast	Contrast
Ólafsfjörður	−.29***	−.22***	−.31***
*Year of survey*			
2009	Contrast	Contrast	Contrast
2012	−.12***	−.08**	.02
*Gender*			
Male	Contrast	Contrast	Contrast
Female	.05	.03	.03
*Age at survey*			
18–25 years old	−.15***	−.15***	−.12***
26–40 years old	−.12**	−.08*	−.11**
41–66 years old	−.05	−.12**	−.07
67 years or older	Contrast	Contrast	Contrast
*Living arrangements*			
Does not live alone	Contrast	Contrast	Contrast
Lives alone	−.04	−.03	.00
*Education*			
Trade certificate	.02	.04	.02
University degree	.05	−.04	.05
Other education	Contrast	Contrast	Contrast
*Employment status*			
Not employed	Contrast	Contrast	Contrast
Employed	−.01	.01	−.02
Adj. R^2^	.11	.07	.11

Finally, a total of 73–74% were rather or very satisfied with the *quality* of health care in both 2009 and 2012, with no significant differences between the 2 surveys (χ^2^=2.0; df=4, p>0.05). Table 3 shows that there is no statistically significant difference in the evaluation of quality of healthcare after controlling for differences in satisfaction between men and women, age, living arrangements, education, employment status and town of residence.

### The results of the interviews

The service users emphasised the fundamental importance of solid primary health care, good local elderly care, some freedom of healthcare choice, and reliable emergency services to be able to live in a rural area like Fjallabyggð.

#### Solid primary health care

All participants emphasised that primary health care, as part of the National Health Care Services, must be located in both towns. The description of satisfactory primary health care was high-quality, effective services with good access, which formed a necessary infrastructure in their community. In Figure 3, this is represented in the microsystem of the model as the service users expressed the need for health services within the community.

Participants experienced that they could always get an appointment with a physician within 24 hours and right away in case of emergencies. Most often, the service was fast and effective. Maria said, *“While we still have primary health care as it is… as long as that is not cut back, we are satisfied”*. The participants asserted that if primary health care were shut down or moved to another municipality, the basis for living in the towns would be gone and the inhabitants would rise up and strongly protest.

Cuts in funding for updating and maintenance of technical equipment in the hospital in Siglufjörður and primary health care meant that people had to travel long distances, for example to Akureyri or further, for services instead of getting them locally. For example, a collection for an X-ray device was arranged and funded by the locals. Jacob said, “*We are now paying for the equipment at the hospital. First, we have to collect money to buy the equipment, and then we have to pay for running them through our taxes. This is regression*”. There is a long and strong tradition in Iceland for clubs and associates to do fundraising for medical equipment, and health care institutions increasingly rely on these groups.

#### Good local elderly care

Service to the elderly was of vital importance to the participants. They felt it was essential to be able to age in place; to live at home or in a retirement home in the local area, have access to hospital care in the last part of life if needed, and have the choice to die in the presence of loved ones. Family members of the elderly also found this very important, referring to the need that elderly care should be provided in the community, or in the microsystem presented in Figure 3.

#### Freedom of health care choice

The tunnel and the union of health care services in both towns increased the possibility for the inhabitants to choose between professionals and locations. This was especially important to people living in Ólafsfjörður where only 1 GP had been practising before the opening of the tunnel. Now there were medical shifts in rotation in the towns and residents drove between the places in 15 minutes. Despite general satisfaction with the tunnels, people mentioned that nature would, however, not change, and that snowstorms and the risk of snow avalanches would still from time to time prevent people from travelling outside Fjallabyggð, their own town. Interpreting this into Bronfenbrenner’s model, seen in Figure 3, all the different environmental levels are in a dynamic interaction with each other based on the attitude and need of the service users. Mistrust and insecurity because of unprofessional attitudes or stagnation was mentioned in exceptional cases. In those occurrences, people drove long distances to get services they liked. Criticism was rarely directed at health care staff personally, rather at their professional competence, and a question was raised as to the continuing education of physicians in rural areas. The participants felt they had a choice regarding medical specialists’ services: either to drive long distances to get the service from a preferred specialist, or to receive it from those visiting their town once or twice a year. The opening of the tunnels shortened the distance for the residents of Siglufjörður to see a specialist in Akureyri.

#### Reliable emergency services

Ambulances, as part of the emergency services, are at the level of the microsystem (Figure 3), as having an ambulance in both towns was considered essential in light of the distance to the next acute hospital; a short reaction time can make the difference between life and death. The importance of helicopters was also mentioned as part of rescue operations and safety at sea. Inhabitants’ trust regarding health professionals was rather strong, influenced by positive professionals’ attitude, conduct and competence. That trust brought the feeling of being safe, even though living in a remote and rather isolated area. The strong bonds between professionals and service users in a small community were mentioned, and connections between inhabitants were likened to a close-knit family. These bonds also brought the feeling of safety, knowing that there is always someone to call upon.

## Discussion

There was significantly less satisfaction with *accessibility* and *variety* of health care services in Fjallabyggð in 2012 after the downsizing of health care services. This meant that service users had to be transferred to the regional hospital in Akureyri, and relatives had to drive long distances in all kinds of weather to visit their loved ones. Some rented a lodging to stay near a sick family member. Interestingly, a high number of inhabitants were satisfied with the accessibility and quality of health care. The reasons for this could be many, and should perhaps be looked more closely in the future with regards to the aspect of personal relationship between service users and health care personnel. In small places where staff is limited and everybody knows each other it might be difficult to criticise the service, or people might just be very pleased with what they have. On top of the downsizing of health care services was the 20% cutback in elderly care compared with a 10% national average [25], seriously threatening users’ expressed will to age in place. The tunnel project allowing, for example, greater access to specialised health care services in Akureyri may have somewhat counterbalanced the downsizing of local health care services, but apparently not enough. The lesson learned from the case of Fjallabyggð is perhaps that before major changes are made, a clear official discussion should take place on how these changes will affect health care service in place, both for the inhabitants and policy makers. The tunnel probably made the downsizing in rural Fjallabygð possible. However, it was never clear where the line between distant specialists and high-tech services versus primary health in the community would be drawn.

Users expressed the fundamental importance of solid primary health care, good local elderly care, having some health care choice, and reliable emergency services to be able to live in a rural area like Fjallabyggð. The participants were adamant about the importance of primary health care. Interestingly, primary health care has been found to be more likely to improve overall equity in health than services directed at specific diseases, because primary care is less costly thus making it possible to share resources more equitably across the population [4,26]. In our research the participants in both towns were satisfied with the short waiting time for local primary health care services, and the perception of *quality* of the health care services was rather high (73–74%) and unchanged in 2012. Most were happy with the attitude of the health care professionals. They trusted the staff, but a question was raised concerning the continuing education of GPs and whether they had opportunity to do so. The Hippocratic Oath taken by GPs stresses the responsibility of enhancing medical knowledge the whole working life. However, because of a lack of GPs in Iceland, time off work for continuing education can be difficult if a replacement is not available. There is a tradition for town councils in rural areas to often hire unexperienced medical students to fill in for GPs when they go on vacation. This is controversial and not practised in neighbouring countries, and claims have been made for reconsidering this tradition [27]. It is known that rural residents sometimes appear to avoid or delay seeking medical assistance [28] and seek such help over longer distances if they are not satisfied with local services [29]. In some cases, people in our study drove long distances to get health care services they liked.

Achievement of health equity requires action on the conditions in which people are born, grow, live, work and age, and on the structural drivers of these conditions globally, regionally, nationally and locally [7]. Even if equity is an important goal of health care, research indicates that health care systems do not deliver health services equitably and that socio-economic differences in both health and health care use may even be increasing [1]. During recent years increasing social differences and inequalities in health have been identified in the Nordic countries [30], and there is evidence of declining support for the unconditional Nordic welfare state, including a “welfare state fatigue” [31]. An example of this in Fjallabyggð is that ambulances were in both towns and the participants found this to be crucial for the safety of the people. Not long ago, there were plans to remove one of the ambulances but the inhabitants strongly protested and the plans were discontinued. Today, however, decisions have been made, despite a growing economy, that rescue team volunteers as opposed to paramedics will operate the ambulance in Ólafsfjörður [32]. The municipal council has protested fiercely, but it is not expected that this decision will be overruled [33].

To be effective, any strategy to promote social justice and equity in health requires a wide-ranging reshaping of policies at all levels and sustained commitment [34]. Sustaining a welfare state is possible and is primarily a matter of determination [29]. Threats to the welfare state should be identified so these can be managed in the service of health-promoting public policies [29]. In times of economic downturn, people who are already most exposed to disadvantage may feel the effects more strongly than do others, health inequity may worsen, and more people may become vulnerable [17]. To ensure equity, health care policymakers should take into account that, beyond population size, the rural population may need more health-promoting efforts and prevention as well as maintained access to health care [17], and that services should be provided as close to home as possible [6]. Reducing health inequalities is in line with human rights and depends on our visions, actions and courage [5]. The issue of inequality in health is a political issue and we have to defend health as a human right continuously [4,10]. Health is a political choice [35], and politicians may seriously underestimate residents’ will to have health care services near to them, even if it may be low tech versus having access to high-tech services much further away.

### Limitations

Residents in rural areas in Iceland constitute a vulnerable population that, in general, report poorer self-related health and yet have lower prevalence of diagnosed diseases, perhaps due to self-migration of those with chronic diseases. Interviews with self-migrants’ concern about health care would have strengthened our case, as from our data we can only speculate about what might happen if an elderly or chronically ill person moved to a larger centre.

Based on the census conducted as part of the project, the estimated response rate was 53% in 2009 and 30% in 2012, which is rather low. Differences in data collection methods are likely responsible for the lower response rate in 2012.

### Conclusions

According to Icelandic law, equal access to health care is a human right that everyone should have irrespective of their place of residence. In Fjallabyggð, a rural area in northern Iceland, serious cutbacks related to the economic collapse have occurred, resulting in downsizing of local health care services. On top of this was the 20% cutback in elderly care compared with the 10% national average, seriously threatening users’ expressed will to age in place. All these major changes are part of a complex social interplay that affects the perspective of health care service users. The tunnel project, providing greater access to specialised health care services in Akureyri, may have somewhat counterbalanced the downsizing in local health care services, but apparently not enough. While this case has rather unique quasi-experimental features, our results have important implications for the provision of health care in rural arctic regions. Users expressed the fundamental importance of solid primary health care, good local elderly care, some freedom in health care choice and reliable emergency services to be able to live in a rural area like Fjallabyggð. Cutbacks may have other effects in rural areas than in more densely populated ones, and should be done carefully and in sustained conversation with the local population. In times of economic downturn, people in rural areas who are already vulnerable may become even more vulnerable and disadvantaged, and the unavailability of certain essential local health care services may lead to self-migration that may again lead to cultural bereavement, changes in identity and concept of self as well as the loss of important social support systems.
